# Measurement of Blood Pressure by Ultrasound—The Applicability of Devices, Algorithms and a View in Local Hemodynamics

**DOI:** 10.3390/diagnostics11122255

**Published:** 2021-12-02

**Authors:** Moritz Meusel, Philipp Wegerich, Berit Bode, Elena Stawschenko, Kristina Kusche-Vihrog, Horst Hellbrück, Hartmut Gehring

**Affiliations:** 1 Department of Cardiology, Angiology and Intensive Care Medicine, University Medical Center Schleswig-Holstein, Campus Luebeck, 23538 Luebeck, Germany; moritz.meusel@uksh.de; 2 Department of Anaesthesiology and Intensive Care Medicine, University Medical Center Schleswig-Holstein, Campus Luebeck, 23538 Luebeck, Germany; wegerich@imt.uni-luebeck.de (P.W.); berit.bode@uksh.de (B.B.); elena.stawschenko@uksh.de (E.S.); 3 Institute of Biomedical Engineering, University of Luebeck, 23562 Luebeck, Germany; 4 Institute of Physiology, University of Luebeck, 23562 Luebeck, Germany; kristina.kuschevihrog@uni-luebeck.de; 5 Department of Electrical Engineering and Computer Science, Technical University of Applied Sciences Luebeck, 23562 Luebeck, Germany; horst.hellbrueck@th-luebeck.de

**Keywords:** blood pressure, measurement, ultrasound, noninvasive, monitoring, arterial, dynamic

## Abstract

Objective: Due to ongoing technical progress, the ultrasonic measurement of blood pressure (BP) as an alternative to oscillometric measurement (NIBP) or the continuous non-invasive arterial pressure method (CNAP) moves further into focus. The US method offers several advantages over NIBP and CNAP, such as deep tissue penetration and the utilization of different arterial locations. Approach: Ten healthy subjects (six female, aged 30.9 ± 4.6 years) volunteered in our investigation. In the ultrasonic BP measurement, we differentiated between the directly measured (pulsatile diastolic and systolic vessel diameter) and indirectly calculated variables at three different artery locations on both arms, with two different ultrasound devices in the transversal and longitudinal directions of the transducer. Simultaneously, NIBP monitoring served as reference BP, while CNAP monitored the steady state condition of the arm under investigation. The Moens–Korteweg algorithm (MKE) and the algorithm of the working group of San Diego (SanD) were selected for the indirectly calculated ultrasonic BP data. Main results: With US, we were able to measure the BP at each selected arterial position. Due to the investigation setup, we found small but significant interactions of the main effects. Bland and Altman analysis revealed that US-BP measurement was similar to NIBP, with superior accuracy when compared to the established CNAP method. In addition, US-BP measurement showed that the measurement accuracy of both arms can be regarded as identical. In a detailed comparison of the selected arterial vascular sections, systematic discrepancies between the right and left arm could be observed. Conclusion: In our pilot study, we measured BP effectively and accurately by US using two different devices. Our findings suggest that ultrasonic BP measurement is an adequate alternative for live and continuous hemodynamic monitoring.

## 1. Introduction

Ultrasonic blood pressure (BP) measurement has become a major focus in the noninvasive assessment of BP [1,2,3,4,5,6,7,8,9,10,11,12,13,14]. Deep tissue penetration, the parallel assessment of the vessel wall dimensions in the longitudinal and transversal directions, and assessment of the blood flow velocity using the Doppler principle are the major advantages of this method. Furthermore, this progressive approach which permits the observation of vessels in deeper vascular sections can provide relevant information on central hemodynamics.

The physical and physiological approaches for assessing arterial BP using ultrasound have been theoretically evaluated elsewhere. Since the physical principle of the ultrasound method cannot measure blood pressure directly, the data for blood pressure can be calculated based on algorithms. In addition to the established methods according to the Moens–Korteweg equation (MKE) [15,16,17,18,19], more current and modified algorithms were used [1,2,3,4,6,10]. These algorithms take following parameters into account: vascular dimensions, blood flow velocity, and congruent pulse waves. Blood flow characteristics associated with the containing blood cells and the associated flow profiles are also relevant, but often neglected in the analysis.

Ultrasonic transducers display information on the local position. The simplicity of handling ultrasound enables rapid changes in transducer position. Alternatively, several flexible transducers can be positioned over relevant vascular sections. Both principles enable the generation of relevant information on local hemodynamics.

The main objective of this study was the extraction of noninvasive information from arterial vessels for the determination of BP at three positions on the left and right arm with ultrasound using two different ultrasound devices. To clarify the universal application, we introduced two ultrasound devices in combination with vessel imaging in the longitudinal and transverse directions. Based on the broad range of algorithms, the MKE [8,13] and the algorithm of the working group of San Diego (SanD) [4,12] were selected as representative. In the examination of healthy volunteers, the oscillometric BP measurement (NIBP) served as a reference. Further, the continuously applicable CNAP^TM^ system also allowed for controlling of a hemodynamic steady state condition along the arm under investigation.

## 2. Materials and Methods

The investigation was conducted in accordance with the Declaration of Helsinki Ethical Principles and Good Clinical Practices, and was approved by the local ethics committee of the University of Lübeck (19-364A). After informed consent was obtained, ten healthy, non-smoking volunteers (four male, six female; age 30.9 ± 4.6 years; all right-handed) were investigated in a relaxed and horizontal supine position on a stretcher under standardized conditions with sufficient access to both arms and without spontaneous movements by the volunteer. The volunteers were asked to abstain from alcohol, caffeine, and taurine as well as intense physical activity for 4 h prior the experiments.

**Figure 1:**
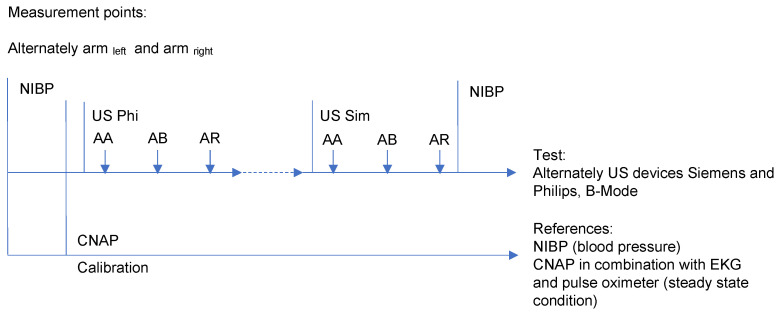
Test protocol of the examination of each subject with essential requirements: (1) Blood pressure is constant. (2) Reference monitor for steady state conditions (CNAP) is on the same arm. (2) Alternate measurements on the left and right arms. (3) Data recording of ECG, pulse oximeter, and CNAP blood pressure curves in near real time.

ECG, pulse oximeter and CNAP^TM^ (Continuous Noninvasive Arterial Pressure; Monitor Dräger Infinity Delta, Drägerwerk AG, Luebeck, Germany) were placed on the first arm (see examination protocol in Figure 1). After a 10-min resting phase, BP was measured oscillometrically three times with a calibrated BP monitor (Modell MTX, Medisana AG, Neuss, Germany) in the reference position at the upper arm (UA; NIBP). Next, the CNAP cuff was installed for calibrating the SmartPod^®^ (Drägerwerk AG, Luebeck, Germany) BP measurements with the cuffs at the UA and as well at the finger site. After removing the CNAP cuff at the UA, the examiner turned the arm a little outwards and started the examination with the ultrasound devices, in each case with the transducer in the longitudinal and transversal directions, and at the three designated positions. At the end of the examination on the first arm, BP was measured three times again via NIBP, controlling the steady state conditions, and data were stored with the Dräger Infinity Monitor. The same examination was repeated on the contralateral arm.

### 2.1. Measurement Systems and Application

The test systems involved two ultrasound devices: a Philips iU22, Transducer L17-5 (17 MHz linear) (Philips Medical Systems, Hamburg, Germany) with software package 6.3.7.745, and a Siemens Acuson S2000, Transducer 14L5 (14 MHz linear) (Siemens Healthcare, Erlangen, Germany) with the software package 400.1.031; both had a frame rate of approximately 30 Hz when using B-mode. The ultrasonic assessment was carried out by a single experienced examiner in order to reduce examination-related errors. Data were stored in a video format, followed by further offline processing.

The noninvasive reference measurements for BP were discontinuously performed with a calibrated oscillometric device and manual data acquisition.

The control for steady state conditions during the examination of one arm included visual and continuous monitoring via Dräger Infinity and CNAP^TM^ SmartPod^®^, and additionally with digital data recordings in near-real time using data software (Data Grabber, license provided by Drägerwerk AG, Medical Division). The beginning and end of the ultrasonic examination phases were marked accordingly. This enabled an almost simultaneous evaluation of the recorded (with CNAP) and generated (with US devices) curves.

CNAP is a method for noninvasive and continuous BP measurement. For this purpose, two finger cuffs are connected to an optical unit. The inflowing blood volume to the finger is kept constant by the cuff and only the pressure pulse is assessed photoplethysmographically. Calibration is performed on the ipsilateral UA using the oscillometric method. The time frame for recalibration can be modified to a maximum of up to 30 min. A calibration can be initiated autonomously by the device during use if a systematic difference is measured between both finger cuffs. This function was switched off for our experiments, in order to have the maximum time frame available. The main advantage of CNAP is the visualization and recording of the arterial BP curve.

The test and reference systems NIBP and CNAP are approved medical products and have a CE mark.

#### 2.1.1. Data Acquisition

Ultimately, the acquisition of 24 data points per test subject resulted in a total of 240 data points for all ten volunteers. At each data point at least 10 to 12 s by ultrasound were recorded in parallel by CNAP and evaluated offline. During the measurement phases on one arm, a prerequisite was that the BP remain nearly constant.

#### 2.1.2. Data Protocol

Ultrasound data acquisition depends on the examiner and the procedure. Timepoints for each measurement were recorded in the reference system (CNAP). The BP (NIBP) was oscillometrically measured three times at the beginning and at the end of each examination, first on the left arm and then on the right arm. Times and measured values were then logged.

### 2.2. Statistical Processing

We worked with a complete factorial examination plan on ten subjects, with the following fixed factors or levels: manufacturer (Siemens | Phillips), arm (left | right), alignment of the transducer (longitudinal | transversal), position of the vessel section (AA | AB | AR). For the subjects, random effects came into the model according to the assumption that individual expected values follow a normal distribution. We estimated the expected values under the various test conditions, their differences, and the 95% confidence intervals from all measurements simultaneously using the mixed model for the analysis of variance (IBM^®^ SPSS^®^ Statistics, Version 27 for Windows, Munich, Germany). This also included estimates for the variance between subjects and residual variance within a subject when the experimental conditions were all the same. The normal distribution was tested with the Q-Q plots of the residuals.

We selected the statistical output as mean values ± standard deviations and confidence intervals. This resulted in a relevant and practice-related presentation of the systematic differences. In addition, we chose the Bland and Altman method to demonstrate the differences between the individual methods, based on the systolic, mean, and diastolic BP. Depending on the results of a systematic trend within the Bland and Altman analysis, the corresponding regression lines and the prediction intervals for the individual data points were calculated and plotted. Finally, we presented the systematic differences between the MKE, SanD and CNAP methods versus NIBP as mean values ± confidence intervals.

#### 2.2.1. Algorithm Based on the Moens–Korteweg Equation (MKE)

The basic assumption when using the MKE was that a small increase in Δ*P* led to an equally small increase in Δ*r*. This assumption requires an elastic vessel and a constant density as well as the associated viscoelasticity of the blood.

(1) $$PWV=\sqrt{\frac{E_{inc}\cdot h}{2r\rho }}\;$$

By inserting the Young’s modulus of elasticity (*E_inc_*)

(2) $$E_{inc}=\frac{\Delta P}{\Delta r/r}\cdot \frac{r}{h}\;$$

into the MKE, the alternating part of the BP curve is calculated according to:

(3) $$\Delta P=\frac{2\cdot PWV^{2}\cdot \;\Delta r\cdot \rho }{r}\;$$

The *PWV* can also be derived from the noninvasively measured BP and the vessel radius (*r*), as well as the vessel expansion (Δ*r*):

(4) $$PWV=\sqrt{\frac{\Delta P\cdot r}{2\cdot \Delta r\cdot \rho \;}}$$

By adding the diastolic BP value from the reference NIBP, the BP can be calculated according to:

(5) $$P=\Delta P+P_{D}\;$$

The derivatives of the formulas are essentially taken from the works of [8,13,20,21].

#### 2.2.2. Algorithm of the Working Group of San Diego (SanD)

The algorithm of the working group of San Diego extracts the values for the BP calculation by measuring the change of the cross-section and, assuming a rotationally symmetrical vessel, thereby derives the change in vessel diameter. Further assumptions are that the vessel is elastic or only slightly changed atherosclerotically, and that the blood viscosity is almost constant. The systolic blood pressure is calculated with:

(6) $$P\;\left(t\right)=P_{D}\cdot e^{\alpha \cdot \left(\frac{A\left(t\right)}{A_{D}}-1\right)}\;$$

In this algorithm, α is described as a rigidity coefficient:

(7) $$\alpha =\frac{A_{D}ln\left(P_{S}/P_{D}\right)}{A_{S}-A_{D}}\;$$

(8) $$A\left(t\right)=\frac{\pi d^{2}\;\left(t\right)}{4}$$

*P_S_* and *P_D_* are added from the NIBP reference.

Algorithm [4] is derived from the investigation by Arndt [14] whereby the relevance of elasticity and rigidity is rather negligible. The authors assume that, given an elastic vessel, the pressure–diameter curve has only a low hysteresis of 0.2%. In the case of atherosclerotically changed vessels, the indicated maximum hysteresis is of 5.2% [4].

### 2.3. Sequence of Video Analysis

**Figure 2:**
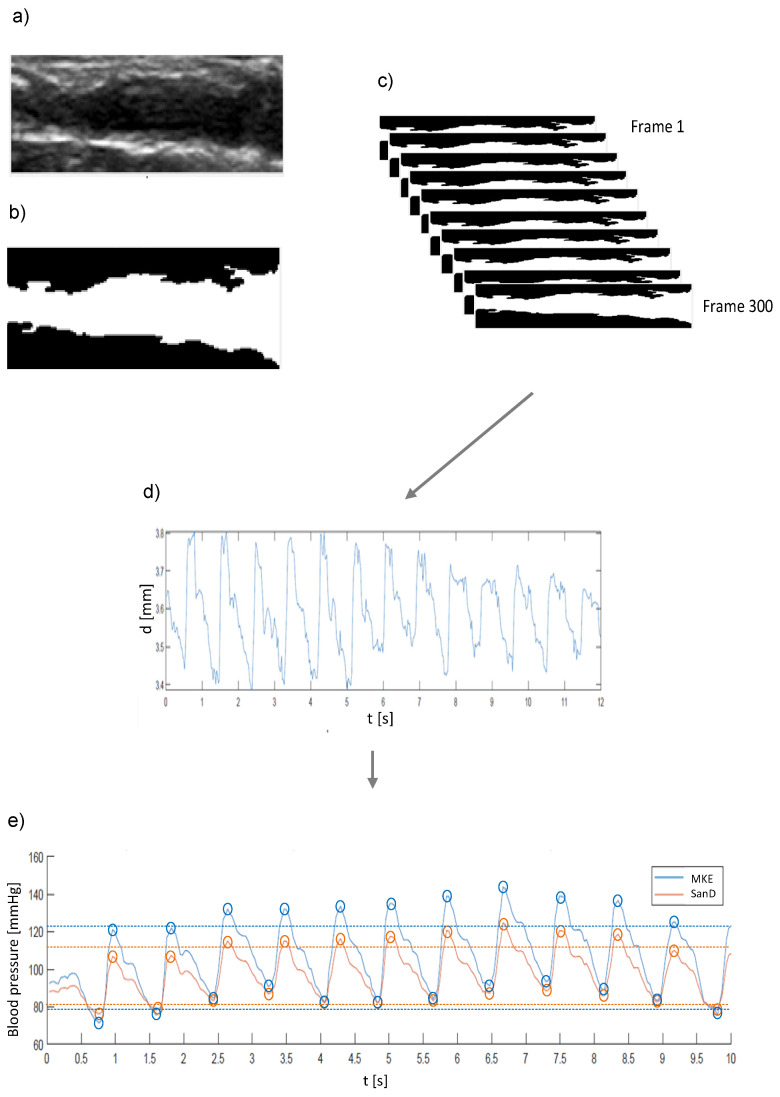
Processing flowchart of the sequential video analysis: (**a**) extraction from the video; (**b**) separation of tissue (black) and vessel area (white); (**c**) calculation of the vessel diameter of approximately 300 frames; (**d**) display as a curve; (**e**) transfer via the algorithm to blood pressure curves over time in seconds.

The basis for the evaluation were video segments of approximately 10 – 12 s in length and with 30 frames per second (fps; see flowchart in Figure 2). A slight variation in fps was accounted for by the software algorithm (MathLab). Based on the vessel selection, the area of interest was cut out (Figure 2a) of the video sequence. After conversion to gray scale and application of a low-pass filter for noise reduction, the area of the vessel could be divided by binarization (Figure 2b), resulting in the separation of vessel (black) and tissue (white). Afterwards, the pixels were counted and divided by the vessel’s length. The vessel diameter was calculated for each individual frame at the selected sequence (Figure 2c). Resulting in a value for the vessel diameter of each frame (d), and, from the vessel diameters of all frames (Figure 2c), the diameter over time results (d (t); Figure 2d).

The parameters of pulse wave velocity (*PWV*, calculated according to [8,13,20,21]) as well as d (t) and ∆d, were part of the MKE algorithm. In contrast, only d (t) and ∆d were included in the application of the algorithm from the working group of San Diego (SanD). After peak detection of *P_S_* and *P_D_* (Figure 2e), further descriptive statistical variables (mean and standard deviations) were calculated.

For calculation of both algorithms, NIBP data served as a reference for calibration.

## 3. Results

Investigating the US devices, we differentiate between the directly measured and the indirectly calculated variables. The first focus was the directly measured variables as an indicator of the displayed precision and thus of the downstream accuracy of the calculated data. The most sensitive parameter to be emphasized is the change in diameter, d (mm), between systole and diastole (Δd_S-D_).

The normal distribution of the data was tested with Q-Q plots of the residuals. Only occasional and minor outliers were found, and accordingly the analysis regarding the confidence intervals was considered sufficient. The offline processing of the ultrasonic signals and the simultaneously recorded curves of the CNAP system failed at 21 measuring points due to unexpected discontinuities in the signals of the CNAP system. Therefore, a total of 219 of the 240 planned measurements were successful.

Corresponding to the significant values in Table 1, one can assume that the directly measured variables are significantly different depending on the vessel diameter at the various examined positions. Furthermore, minor but systematic differences can be expected between the left and right arm in this series of tests, since all volunteers were right-handed. The BP values calculated from the ultrasound data showed a significant shift regarding the ultrasound device used. However, it is essential that the calculations of BP values are independent of the measurement position on the arm.

Comprehensive and systematic deviations between the examinations with both US devices and the alignment of the transducers in the longitudinal and transversal directions, both for the right and for the left arm and in relation to the selected positions, can be ruled out. There are minor but systematic differences in both the alignment of the transducer and in the US systems, which are most evident in the differences in the diameter (Δd_S-D_). However, regardless of these findings, a difference in hemodynamics between the left and right arm is visible at position AB and AR, as these are captured both by the alignment and the US systems.

**Figure 3:**
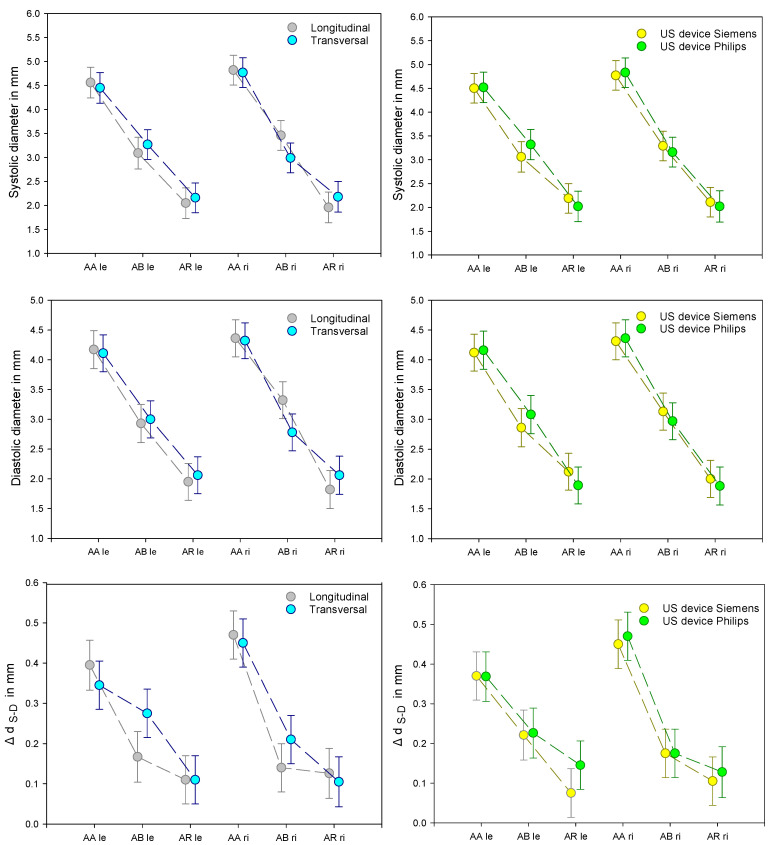
Course of the variables recorded directly with ultrasound (d_S_, d_D_, and Δd_S-D_; d = diameter), measured in longitudinal and transversal alignment on the left side and with US devices from Siemens and Philips on the right side; each at the three selected measuring points A. axillaris (AA), A. brachialis (AB) and A. radialis (AR), specified for the left and right arm (x axis). Statistical significances are not entered in this overview. To delimit relevant differences, the mean values and the confidence intervals were selected.

The corresponding mean values and confidence intervals are summarized in Figure 3.

BP values were calculated according to the MKE and the SanD algorithms, and therefore represent indirectly derived variables. The agreement between the selected methods is shown as a mean value ± standard deviation in Table 2, taking into account the alignment of the transducers and the US systems used. The MKE and SanD algorithms are nearly equivalent to the values of the NIBP reference method and the CNAP system. There are significant differences depending on the measurement position. However, this should be interpreted in the sense of a “practical significance” compared to the small deviation.

**Figure 4:**
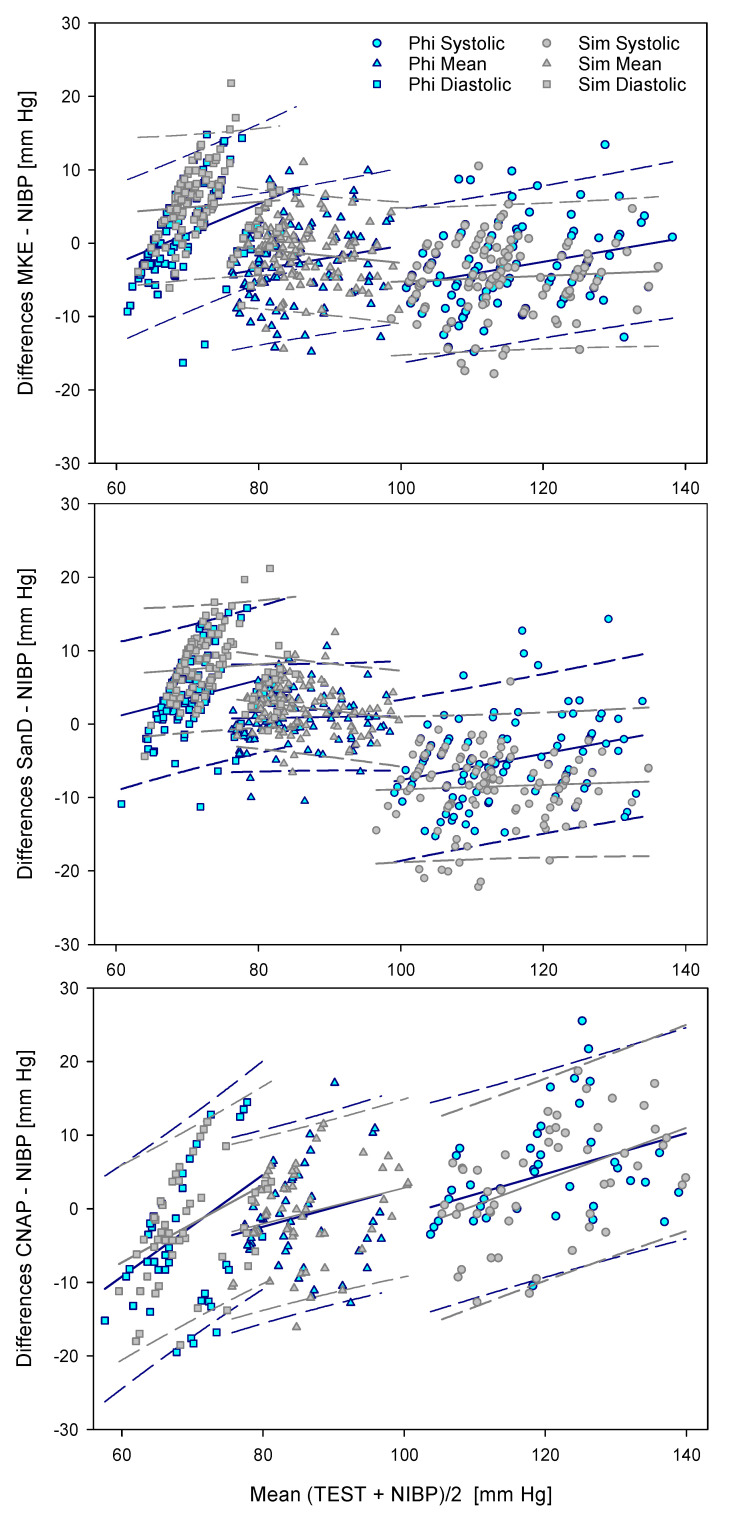
Plots according to Bland and Altman. An entry of bias and limits of agreement was dispensed with in favor of regressions (solid lines) and prediction intervals (mean dotted lines).

For the systematic assessment of the differences, the analyses according to Bland and Altman are presented in Table 3. The deviations of the BP values calculated with US according to MKE and SanD are lower than for the CNAP method. Both MKE and SanD overestimate diastolic and underestimate systolic BP, while the opposite is apparent for the CNAP procedure (Figure 4).

Since there is a small but significant linear dependency in the data configurations (Table 4), the direct results of Bland and Altman should be handled with care [24]. Accordingly, further diagrams integrate the regression lines of the mean data and the prediction intervals for each data point separated for the devices [25,26,27].

The data plots of the CNAP system also show some small differences between the US devices, although they were not directly involved here.

## 4. Discussion

The main objectives of this study were the extraction of noninvasive information from arterial vessels in order to determine BP at three different positions on the left and right arm by ultrasound. To clarify the universal applicability, we investigated two different ultrasound devices with longitudinal and transversal transducer directions. Based on the broad range of algorithms, the Moens–Korteweg equation (MKE) and the algorithm of the working group of San Diego (SanD) were selected as representative.

The main results of the present study are that both US systems with transducer alignment in longitudinal and transversal directions generated similar information to NIBP. Furthermore, the ultrasonic method can be applied at three different positions on both arms with congruent results. Both algorithms used to determine BP, MKE and SanD, provide reproducible data with an accuracy that is superior to the validated CNAP method when compared to NIBP.

The data collected from all three measuring points at the respective extremity indicate a small but systematic difference between the AA and AB, as well as between the right and left arm. In brief, the Δd_S-D_ of the right arm shows a different course in contrast to the left arm (Figure 4). Therefore, by interpreting our data, a methodological effect within the study protocol as well as an individual effect should be considered, and our data should be assessed with appropriate caution.

### 4.1. Characteristic Features of US Based BP Measurement

The advantages of US are various: signals can be generated with high resolution and rapid imaging sequences. Direct, pulse-shaped wall movements and spatial dimensions can be recorded. US further enables recording of deeper vessel sections, and thus allows data acquisition from more centrally located arteries such as the external and internal carotid arteries.

Simultaneously, PW Doppler analysis can be used to measure flow velocity and to determine the local pulse wave velocity (*PWV*). A parallel image recording of B-mode and PW-Doppler has not yet been implemented by default in the software of the US device manufacturers, as favored by Rabben [8]. Accordingly, the *PWV* in the present study has been calculated by further application of the MKE and the oscillometrically measured BP. If the parallel recording becomes routinely available, the *PWV* calculation can be implemented directly in the ultrasonic measurement of BP. In addition, it is possible to obtain information about blood flow using specific spatial dimensions, which provides the possibility of further measurement by US.

However, the ultrasonic assessment via handheld transducers allows only punctual measurements of selected vessel sections. Further disadvantages include inhomogeneous skin contact even when US gel is applied as well as the rigidity of the linear transducer surface. In addition, if pressure is applied to the examined artery, a falsification of the target variable (BP) is possible. These downsides of rigid handheld US transducers have led to the development of flat, flexible, and stick-on US pads [4]. US pads allow direct positioning over the relevant vessel sections (arterial and venous) and continuous data acquisition at multiple locations.

The aforementioned disadvantages and possible sources of errors cannot be entirely ruled out by an experienced investigator, but were largely avoided. Since even slight exertions of pressure by the transducer can lead to changes in the pulse curve, we continuously monitored acute BP alterations with the CNAP system. The simultaneous CNAP measurement showed no acute ipsilateral reduction in the target variable BP due to arterial compression by the US transducer throughout the experiments.

### 4.2. Characteristic Features Regarding Algorithms and Calibration

The wide range of US-based methods for BP measurement can be divided into four groups:

The first group works according to the physiological principles of Moens [28] and Korteweg [29], Young’s modulus of elasticity [30], and the Bramwell–Hill model [31]; the common link is the *PWV*, which depends on the blood pressure and blood volume flow, as well as the elasticity of the examined vessel and the viscosity of the blood.

By simplification of some assumptions and paradigms of the first group (e.g., circular vessel, negligible viscoelasticity), the second group calculates BP through variables which can be measured directly in B-mode [4]. The process is referred to as “wall tracking” [32].

The algorithms of both groups require external calibration by at least one systolic and/or diastolic blood pressure value.

The third group contains the next level of simplification, measurement without an external calibration value. Nabeel [10] uses two rapidly successive measuring points in the early systolic phase as basis of his mathematical model. Subsequently, a ratio is calculated which represents a value for the “stiffness index”, *β*. Supposing that the stiffness index *β* is constant in the early systolic period, several variables can be extracted, ultimately resulting in the “level of diastolic blood pressure” value. The working group of Zakrzewski [1] applied a defined and measured contact pressure with the US transducer and used this pressure change and the corresponding deformation of the artery wall as an internal measure for the calibration (“force driven method”).

As a fourth group, one might consider continuous measurement via flexible sensors in several positions [4,9,12].

The MKE and SanD algorithms require calibration through a direct measuring method. For investigation on healthy volunteers, noninvasive, oscillometrical BP measurement is the method of choice, and was therefore used as reference in the present study. The NIBP defines the limits of the US-based BP measurement. While the values of the directly measured BP are included in the SanD algorithm, the application of MKE requires additional integration of the *PWV*. The fact that *PWV* is not measured directly but rather indirectly through repeated application of MKE, and thus calculated from the NIBP, shows a clear limitation of our study. The main reason for this limitation can be found in the US devices used in our experiments. Parallel measurement of the local *PWV* by US (B-mode plus Doppler-mode) offers a promising approach and has already been confirmed experimentally [8], but is not yet available on all standard US devices. An alternative to recording the local PVW is the additional use of optical or magnetic sensors, e.g., B. Nabeel et al. [10], who measured the local *PWV* via plethysmography in parallel to US and integrated this data in an algorithm derived from the Bramwell–Hill model.

### 4.3. Characteristic Features Regarding Blood Pressure Measurement, Medical Application and Requirements, and Reference Methods

Nothing is as divergent as the definition of BP on the one hand and the methods for BP measurement in different areas of application on the other hand [33,34].

The physiological derivation on which BP values are measured is controversially discussed. The systolic BP is subject to modulation by the cardiac stroke volume and elastic properties of the vessel [35]. The MAP, derived from systolic and diastolic BP when measured noninvasively, is often used as a threshold for therapeutic interventions in order to maintain adequate organ perfusion.

There are three possible clinical scenarios for BP measurement: First, the hypertensive patient with associated cardiovascular diseases; second, the normotensive patient; and third, the hypotensive patient, which further includes patients who are undergoing operations with general anesthesia. The detection of BP drops and the duration of hypotonic phases is essential to sufficiently avoid organ damage in anesthetized patients. Quick adaptation to the patient’s individual BP during anesthesia reduces the risk of organ dysfunction [36]. Hence, even a close-meshed but discontinuous BP measurement can be insufficient in these patients. The following BP thresholds have been defined: a decrease in systolic BP < 80 mmHg or <20–30% of the initial value [37] and/or a decrease in MAP < 65 mmHg or <20% of the initial value [35,38]. Ergo, there is a clear demand for precise, continuous, and noninvasive BP measurement methods to detect a BP ≥ 60 mmHg with an accuracy of ±5%, corresponding to ±3 mmHg.

### 4.4. CNAP as a Reference for Performance on Test Arm

The CNAP system provides BP data with “acceptable” [39,40], “comparable” [41] and “clinical useful” [42,43] precision and accuracy, as well as with a “larger discrepancy than defined as acceptable” [44] when compared to NIBP and invasive BP measurement. In addition, there is a “lack of evidence” of accuracy in CNAP’s BP data [45].

CNAP is a noninvasive and continuous measuring device, and currently fills the gap in BP monitoring. Due to the above-mentioned limitations, it might support the perioperative BP management for anesthetized and hemodynamically stable patients, and can potentially reduce severe periods of hypotension. The CNAP monitor provides a BP curve in near real time and therefore allows the assessment of beat-to-beat performance.

**Figure 5:**
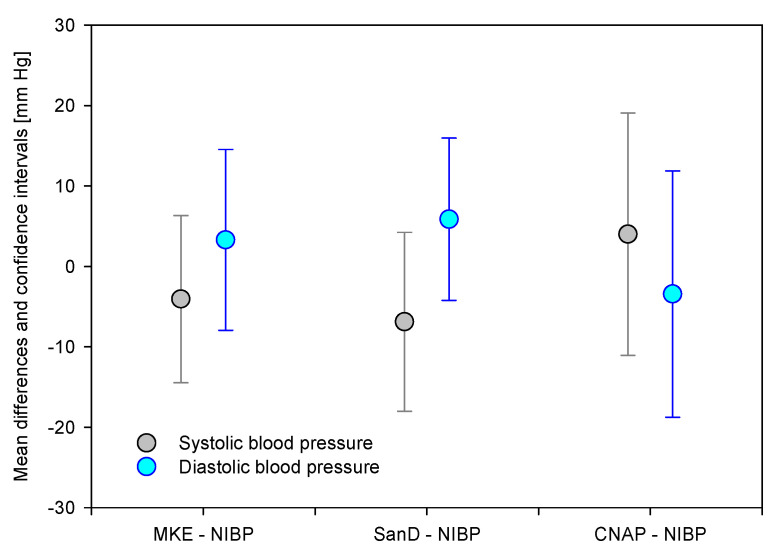
Comparison of the differences (test–ref) in blood pressure as mean ± confidence intervals for MKE, SanD and CNAP and related to the systolic and diastolic blood pressure.

During our experiments, the BP curve of the CNAP system was visually checked for consistency. In addition, continuous recordings of the CNAP data were checked offline and evaluated to the corresponding data points. In this signal analysis, sudden alterations (>5% of the initial value) of the CNAP system occurred in 21 of the 240 measuring points. To ensure a balanced analysis, these data points were excluded from analysis. Despite this adjustment, CNAP differed from NIBP on a larger scale (see also Table 2 and Table 3, and Figure 5), i.e., CNAP underestimated the diastolic and overestimated the systolic BP.

### 4.5. NIBP as a Reference

BP monitoring during anesthesia is essentially based on NIBP [46,47,48,49]. An intra-arterial pressure measurement is necessary in critically ill patients.

In the present study, the oscillometric BP measurement on the upper arm was chosen as a reference. To improve the accuracy of NIBP, the measurement was repeated three times and the mean value was regarded as the reference value. This further allows a statement about the consistency during the ultrasonic examination, given that the measurement is repeated on the same arm after the examination and the patient is in a steady position.

With this set up, we did not choose the “gold standard” [46], but rather a reference value that can be safely handled within the setup. This selected reference method may differ from the “gold standard”, but can be considered equivalent [50,51,52].

The use of NIBP to estimate intra-arterial pressure is controversial. Invasive intra-arterial pressure measurement represents the reference method for critically ill patients, since it provides a continuous BP monitoring [53]. However, several vascular and technical features can lead to measurement errors [54,55]. In addition, the risk of intra-arterial BP measurement via catheter for volunteers must also be considered.

The main point of criticism is the variability of measurement accuracy. However, this applies equally to other BP measuring methods, intra-arterial measurement as well as auscultatory and oscillometrical measurement. This variability in measurement accuracy has been the subject of review articles [56]. It should be emphasized that no system is systematically highlighted.

Our results suggest that US-based methods are applicable for BP measurement in healthy subjects. Further clinical studies are needed in order to confirm our findings.

## 5. Limitations

### 5.1. Methods

For our study we examined discontinuous BP measured via ultrasound, and used NIPB as a reference. Since we used CNAP as a continuous reference in measurement along the investigated arm, we encountered minor protocol violations due to CNAP calibration. This ultimately led to the loss of 21 of 240 data points, which were excluded from analysis.

The behavior of CNAP in this regard is known. Particularly in critical BP changes or differences between both finger cuffs, the system usually starts a complex and long-lasting oscillometric calibration with the upper arm cuff, and thus interferes with simultaneous BP assessment. The measurement accuracy of this method is somewhat reduced compared to the discontinuous NIBP measurement.

Since we needed the *PWV* for calculating the BP with MKE, a second approach by MKE with NIBP was required. This would certainly have a systematic error as a consequence. However, the data presented suggests that our approach is applicable. For future clinical experiments, it should be considered that direct measurement of the local *PWV* might show more stable results.

The limitations in the use of NIBP as a noninvasive reference for BP will continue to be a focus of discussion in future studies. Both NIBP as well as the intra-arterial and invasive BP measurement have pros and cons. In order to solve the discussion, US-based methods could bring more information.

#### 5.1.1. Test with Volunteers

A small cohort of ten subjects undergoing the examination has several statistical limitations.

The first limitation is the assumption of a random distribution of the effects through the selection of the subjects. Secondly, the high number of data points, which is responsible for a quickly visible significance in the presence of only minor clinical differences or abnormalities.

Finally, the Bland and Altman analysis as a recognized method for comparing measurement accuracy between two methods is limited if clear trends can be found. We introduced an extended analysis at this point, which also allows a direct assessment through the visual graphical representation of all data points.

#### 5.1.2. Algorithm

Two disadvantages of the US and the algorithms we used are the exclusion of elasticity of the vascular wall and the viscoelasticity of the blood. The assumption is that the change in the elasticity of the vascular wall in advancing arteriosclerosis as well as the variable of the viscoelasticity of the flowing blood are only rudimentarily considered in the algorithms. In modern patient BP management prior to and during operations and interventions, certain changes in viscoelasticity in case of blood loss with therapeutic volume application and significantly reduced number of erythrocytes and hemoglobin concentration may occur.

It can be assumed that in future clinical investigations and based on further technical or software developments data from US imaging, both for measurements in B-mode and for Doppler signals, could also be extracted and incorporated into the resulting analysis in a modulating way.

### 5.2. In Summary

The following results were obtained from this study on healthy volunteers without known cardiovascular diseases.

The determination of BP with ultrasound is successful regardless of the US system used and the alignment of the ultrasonic probe. The CNAP device is a general reference method for measuring continuous BP in both arms.

The algorithms MKE and SanD are calibrated with the NIBP values. These form a kind of framework and define the limits. It is therefore understandable that the data according to MKE and SanD are within these limits. In this study setup they overestimate the diastolic and underestimate the systolic BP.

The CNAP calibration with the upper arm cuff requires a maximum 30 min interval. A shorter calibration time could not be set up due to the course of the investigation. This may generate a systematic error by using the upper arm cuff, both per se as well as through the long calibration interval.

A relevant difference between MKE and SanD is the variable of the *PWV*, which can be found squared in MKE. This point is clinically relevant when BP drops significantly. In this area of a hypotonic circulatory reaction, the local *PWV*, if measured directly, can then be used as a further variable, and additionally provide a reliable quantification of the hypotension.

Relevant variables generally find entrance in these algorithms regarding the vascular dimensions, blood flow and pulse waves, and associated velocities. The flow characteristics of the blood in connection with the blood cells in it and the associated flow profiles are also relevant, but are often neglected in the analysis.

In addition to a continuous measurement that can only be obtained on a calm level, information about the “dynamic cardiovascular status” will be necessary in the future. This means the application at a reference level to the standard approach (upper axillary fold) and under the conditions of movement and physical exertion. This is a challenge for the measurement process with regard to artifact reduction and signal quality. At the same time, however, the clinical aspect of measuring BP in the event of a significant reduction in BP (severe hypotension) and its use in young and youngest patients must also be taken into account.

An increasing subject of clinical considerations is the complexity of the status of central and peripheral vessels, to be emphasized as a mismatch of stiffness, elasticity and increasing resistance [57]. Since ultrasound enables the assessment of more centrally located arteries, e.g., the A. carotis [32], more detailed and reliable information can be expected here in the future.

Ultrasonic transducers only display information on the local position. The simplicity of handling ultrasound transducers means that rapid changes in position can be set up, or alternatively, that several flexible transducers can be positioned over the relevant vascular sections. Both considerations have in common that the possibility is shown here to generate relevant information on local hemodynamics.

The value of US-based methods and of the algorithms becomes relevant when data can be measured in patients during the onset of severe hypotonic phases with and without intravascular volume depletion. It is during these phases that measurement of local *PWV* and intravascular blood flow in the vessel at the measurement site can provide clinically relevant information. Only if this can be achieved can the recommendation be made to use US-based methods for BP determination. The advantages far outweigh the previous disadvantages. These disadvantages can be transformed into clinically applicable advantages through technical and methodological advancements.

## 6. Conclusions

This pilot study of BP measurement with ultrasound delivers insight into several selected methods: two clinical standard devices, alignment of the transducer, and the two algorithms used. Due to the large amount of data in ten subjects, we found small systematic differences with slight confidence intervals. Both US-based methods show value in the range of the NIBP and CNAP system, with superior accuracy compared to CNAP. Having in mind the perspective of making ultrasound BP measurements at selected, hemodynamically relevant sites, it seems to be a possibility in the future to enable continuous and noninvasive monitoring of essential hemodynamic parameters.

## Figures and Tables

**Table 1:** Output of the complete factorial examination plan on ten subjects with 219 data points, the relevant parameters, test of main effects (interactions), and significances (*p*).

Directly Measured Variables by Ultrasound			L vs. T	Sim vs. Phi	Le vs. Ri	Position
Diameter	Δd_S-D_	*p*	0.610	0.274	0.871	0.000
	d_S_	*p*	0.576	0.962	0.469	0.000
	d_D_	*p*	0.642	0.759	0.523	0.000
Directly measured blood pressure variables						
NIBP	*P_S_*	*p*	0.232	0.409	0.000	0.786
	P_M_	*p*	0.376	0.914	0.000	0.854
	*P_D_*	*p*	0.830	0.549	0.000	0.935
CNAP	*P_S_*	*p*	0.955	0.081	0.000	0.098
	P_M_	*p*	0.886	0.703	0.000	0.581
	*P_D_*	*p*	0.882	0.161	0.000	0.632
Calculated blood pressure variables						
MKE	*P_S_*	*p*	0.021	0.030	0.980	0.553
	P_M_	*p*	0.105	0.000	0.511	0.738
	*P_D_*	*p*	0.547	0.000	0.422	0.959
SanD	*P_S_*	*p*	0.000	0.000	0.341	0.837
	P_M_	*p*	0.127	0.002	0.478	0.646
	*P_D_*	*p*	0.592	0.000	0.154	0.659
			*p* < 0.05			

**Table 2:** Indirectly calculated values for BP according to MKE and SanD, as well as the directly measured values for BP with CNAP and NIBP. The first group contains the values for all measurement times (ALL). In the following groups the values are sorted for the arrangement of the transducer (longitudinally and transversally), for the US devices used (SI = Siemens with 14 MHz transducer and PHI = Philips with 17 MHz transducer), as well as the examinations on the left and right arms.

Tested Procedures		MKE			SanD			CNAP			NIBP		
[in mm Hg]		Mean	sd	n	Mean	sd	n	Mean	sd	n	Mean	sd	n
All	*P_S_*	112.9	9.67	219	110.1	9.83	219	121.0	11.60	219	116.9	8.76	219
	P_M_	86.2	6.12	219	87.0	5.91	219	84.4	7.08	219	85.4	5.83	219
	*P_D_*	72.9	6.37	219	75.4	5.10	219	66.1	7.43	219	69.6	4.96	219
Longitudinal	*P_S_*	112.0	10.00	108	108.7	9.00	108	120.8	11.60	108	116.7	8.50	108
	P_M_	85.8	6.29	108	86.6	5.84	108	84.3	7.04	108	85.3	5.71	108
	*P_D_*	72.7	6.59	108	75.6	6.00	108	66.2	7.40	108	69.6	4.91	108
Transversal	*P_S_*	113.9	9.27	111	111.5	9.50	111	118.9	11.70	111	117.2	9.04	111
	P_M_	86.6	5.96	111	87.4	5.97	111	84.5	7.15	111	85.5	5.98	111
	*P_D_*	73.0	6.17	111	74.2	6.01	111	66.1	7.49	111	69.0	5.02	111
Siemens	*P_S_*	112.4	9.00	114	106.7	8.94	114	120.5	11.91	114	116.9	8.70	114
	P_M_	87.2	5.30	114	87.7	5.63	114	84.6	7.06	114	84.4	5.83	114
	*P_D_*	74.7	5.26	114	77.4	5.26	114	66.7	7.20	114	68.7	4.97	114
Philips	*P_S_*	113.6	10.34	105	111.9	10.47	105	121.4	11.34	105	116.9	8.87	105
	P_M_	85.1	6.78	105	86.2	6.12	105	84.1	7.13	105	84.2	5.87	105
	*P_D_*	70.9	6.87	105	72.2	6.07	105	65.4	7.66	105	69.5	4.97	105
Left arm	*P_S_*	112.4	8.71	109	108.9	9.48	109	118.7	11.54	109	115.7	7.42	109
	P_M_	85.7	5.80	109	86.4	5.46	109	81.6	6.34	109	84.1	4.98	109
	*P_D_*	72.3	6.38	109	74.7	5.84	109	63.0	6.76	109	68.3	4.27	109
Right arm	*P_S_*	113.5	10.54	110	108.4	10.21	110	123.2	11.30	110	118.2	7.42	110
	P_M_	86.8	6.41	110	87.5	6.30	110	87.2	6.70	110	86.7	6.34	110
	*P_D_*	7.3	6.34	110	75.0	6.08	110	69.1	6.82	110	70.8	5.28	110

**Table 3:** Analysis according to Bland and Altman [22,23] for the test procedure against the reference NIBP. Bias = test - ref; Precision = ±1 sd; Limits of Agreement (LoA) = ±1.96 sd.

[mm Hg]		MKE-NIBP	SanD-NIBP	CNAP-NIBP
*P_S_*	Bias	−4.02	−6.86	3.99
	Precision	5.21	5.57	7.58
	LoA	10.41	11.13	15.15
	n	219	219	219
P_M_	Bias	−2.04	1.62	−1.00
	Precision	4.70	3.50	6.35
	LoA	9.39	6.99	12.70
	n	219	219	219
*P_D_*	Bias	3.27	5.86	−3.51
	Precision	5.64	5.06	7.67
	LoA	11.28	10.12	15.34
	n	219	219	219

**Table 4:** The R^2^ values for the regressions in Figure 4, demonstrating a small but systematically linear dependency. * marks the group of the CNAP data, which is independent from the US devices.

US Device	Siemens	Philips
	R^2^ values	R^2^ values
*P_S_* MKE	0.004	0.080
P_M_ MKE	0.015	0.034
*P_D_* MKE	0.005	0.139
*P_S_* SanD	0.003	0.086
P_M_ SanD	0.034	0.001
*P_D_* SanD	0.006	0.059
*P_S_* CNAP *	0.198	0.123
P_M_ CNAP *	0.053	0.047
*P_D_* CNAP *	0.143	0.172

## Abbreviations

α	rigidity coefficient
A	cross-sectional area
AA	Arteria axillaris
AB	Arteria brachialis
AR	Arteria radialis
B & A	Bland and Altman
BP	blood pressure
CNAP	continuous noninvasive arterial pressure
d	diameter (in mm)
D	diastolic
dD	diastolic diameter
Δd_S-D_	difference dS-dD
DS	systolic diameter
ECG	electrocardiogram
E_inc_	Youngs elastic modulus
fps	frames per second
h	wall thickness
Le	left
L	longitudinal
M	mean pressure
MKE	Moens Korteweg Equation
NIBP	noninvasive blood pressure
P	pressure (in mm Hg)
PD	diastolic pressure
Phi	Philips
PM	mean pressure
PO	pulse oximeter
PS	systolic pressure
PWV	pulse wave velocity
r	arterial radius (in mm)
Ri	right
S	systolic
SanD	algorithm of San Diego
Sim	Siemens
T	transversal
UA	upper arm
US	ultrasound
V	blood volume
v	velocity
Δr	arterial dilatation
ρ	density (blood)
